# Food service safety and hygiene factors: a longitudinal study on the Brazilian consumer perception

**DOI:** 10.3389/fnut.2024.1416554

**Published:** 2024-10-31

**Authors:** Natália Caldeira de Carvalho, Clarisse Lolli e Silva, Juliana Costa Liboredo

**Affiliations:** ^1^Department of Food, Universidade Federal de Ouro Preto, Ouro Preto, Brazil; ^2^Department of Nutrition, Universidade Federal de Minas Gerais, Belo Horizonte, Brazil

**Keywords:** food service, safety, hygiene, consumer, ready-to-eat food, COVID-19

## Abstract

The objective of this study was to investigate the perceptions and attitudes of consumers toward food service safety and hygiene when purchasing ready-to-eat food. Data were collected at three time points: before (T0) and during the COVID-19 pandemic (T1 and T2). Among the 333 participants, 45.9% reported fear of contracting COVID-19 when purchasing off-site meals, and 78.4% for on-site meals in T1, compared to 21.0 and 52.0% in T2, respectively (*p* < 0.001). Hygiene and cleanliness of the establishment became less important for participants when selecting food services throughout the pandemic (T0: 42.6%; T1: 41.1%; T2: 0.0%; *p* < 0.01). Security protocols during off-site and on-site purchases were considered important by more participants in T1 (47.7 and 27.6%, respectively) than in T0 (28.8 and 9.0%, respectively), with a decrease in T2 (0 and 16.5%, respectively; *p* < 0.01). Regarding food delivery services, concerns about hygiene decreased in T1 (44%) compared to T0 (63.7%) but increased again in T2 (76%; *p* < 0.01). Precautions with the food packaging was less prevalent at least during one point in the pandemic compared to T0 (*p* < 0.01), while heating food before consumption was more common at the onset of the pandemic (T1) but declined by T2 (*p* < 0.01). Furthermore, the use of cash decreased while contactless payment methods increased during the pandemic. In conclusion, different phases of the COVID-19 pandemic significantly influenced consumer behavior and attitudes toward purchasing ready-to-eat food.

## Introduction

Consumers are influenced by various factors when selecting food service options, including food prices, safety, traceability, place of purchase, frequency of consumption, distance, and sensory attributes of food (1). Their decisions regarding food safety are based on both behavioral and cognitive factors, such as culture, beliefs, risk perceptions, emotions, trust, consequences, and predominantly, external characteristics of the restaurant (2, 3). A meta-analytic review (4) identified key determinants of food safety risk perception, including socioeconomic status, subjective characteristics, knowledge, and trust. While knowledge is essential for promoting food safety, because it can promote positive attitudes, it is often insufficient alone to change practices (5). Trust in government, media, manufacturers, retailers, and certification entities also positively influences risk perception (1). However, in Brazil, consumers tend to distrust the institutions responsible for ensuring food safety (6).

Zanetta et al. (2) found that previous experiences, such as a foodborne illnesses, specific public incidents, and food safety crisis, increase risk perception and protective behaviors. Although SARS-CoV-2 is not transmitted through food intake or contact with food packages, studies during the COVID-19 pandemic identified greater consumer concern regarding hygiene practices and food safety (7–12). The COVID-19 pandemic underscored the importance of food security (13) and altered the dynamics of the food service industry (14). Food service establishments were compelled to review, enhance, and implement good hygiene practices and food safety management systems to mitigate the spread of SARS-CoV-2 (15, 16). Concurrently, consumers became less inclined to dine in restaurants (on-site consumption) due to concerns about contamination and/or establishment closures (6, 17), leading to an increase in the use of food delivery services (off-site consumption) (18, 19).

These pandemic-related concerns and behavioral changes may persist even after stabilization (11). To date, these impacts and behavioral changes have not yet been comprehensively evaluated. Food security is not possible without food safety. A better understanding of the attitudes, concerns, and behaviors of consumers when purchasing and consuming food away from home could guide governmental actions aimed at improving public trust and autonomy regarding food safety, especially as concerns about food risks continue to grow.

Therefore, this study had two objectives: (a) to identify changes in the consumption of home-cooked meals and ready-to-eat food from food services; and (b) to reveal the perception of consumers of food service safety and hygiene factors by evaluating their fears of contracting COVID-19 from ready-to-eat food consumption, the factors considered important to choose a food service, their main concerns and care when purchasing food delivery, and the payment methods used.

## Methods

### Study design

This longitudinal study was conducted using an online questionnaire administered at two points during the COVID-19 pandemic. The first phase took place from September 21 to October 28, 2020, approximately 6 months after the onset of the pandemic. At this stage, participants provided information about their consumption behavior before (T0) and during the pandemic (T1). The second phase occurred from April 27 to June 27, 2022, approximately 18 months after T1, and the participants reported their consumption behavior at that time (T2).

The study adhered to the Declaration of Helsinki and received approval from the Research Ethics Committee of the Federal University of Ouro Preto (number 34.335.120.0.0000.5150).

### Participant recruitment

A convenience sample of adults (aged 18 years or older) with Brazilian nationality was employed. Incomplete questionnaires and responses from participants under 18 were excluded. In the first phase, snowball sampling was used to recruit participants by distributing the survey link via email and social media platforms (Instagram, Facebook, LinkedIn, and WhatsApp) to the general public residing in Brazil. The same participants were invited to take part in the second phase through the email provided in the first phase.

### Questionnaire

The questionnaire was designed by the researchers and pretested by 10 individuals to refine and adjust the questions (Supplementary material). Volunteers took approximately 15 min to complete the questionnaire. The questions were based on market research (20) and a review of the literature regarding consumption behavior related to food delivery and safety measures to limit the spread of COVID-19 (21–27). Participants were directed to the questionnaire after agreeing to participate. The questionnaire was divided into three sections: sociodemographic characteristics; frequency of home-prepared meals, on-site, and off-site (delivery, take-away, drive-thru) services; and food service safety and hygiene factors in consumer perception.

Sociodemographic characteristics included age, gender, hometown size, education level, marital status, household size, and *per capita* income. The section on frequency of home-prepared meals and on-site and off-site services included questions about breakfast, lunch, and dinner. For each question, participants selected a frequency from predefined options, which were assigned numerical values to facilitate the assessment of changes in consumption frequency among the periods studied (between T0 and T1, and between T1 and T2). The frequency of consumption was coded as follows: 0 for “never,” 0.5 for “rarely,” 1 for “once a week,” 3 for “2–4 times/week,” 5.5 for “5–6 times/week,” and 7 for “once a day.” The frequency at T0 was subtracted from the frequency at T1, and the frequency at T1 was subtracted from the frequency at T2 for each participant. A positive difference indicated an increase in the frequency of consumption between the evaluated time points, while a negative difference indicated a decrease [adapted from Liboredo et al. (18)].

In the section regarding food service safety and hygiene factors, participants were asked in T1 and T2 whether they were afraid of contracting COVID-19 from on-site and/or off-site services. These questions used a five-point Likert scale (1-strongly disagree; 2-disagree; 3-neither agree nor disagree; 4-agree; 5-strongly agree). A fear of contracting COVID-19 was indicated by responses 4 or 5, while participants who selected option 3 were considered neutral, and those who chose 1 or 2 were classified as not afraid. Participants were also asked about factors influencing their food service selection, their concerns and care when using off-site services, and the payment methods used during the purchase of ready-to-eat food from food services. These questions were asked in relation to T0, T1, and T2. Among the factors influencing food service selection, the option “hygiene and cleanliness of the establishment” was included. Although cleanliness is technically a component of hygiene, the terms may have distinct meanings in popular usage. Therefore, both terms were included in the answer options. Cleanliness was defined as the condition of being free from filth, dust, and other impurities and was associated with the physical appearance of the space. On the other hand, hygiene encompassed practices and conditions that promote health and prevent disease, including maintaining cleanliness to ensure a safe and healthy environment.

### Statistical analysis

Results were presented as absolute numbers and frequencies. The McNemar Test and Cochran’s Q test were used to compare paired samples of categorical variables in the two (T1 and T2) and three time points studied (T0, T1, and T2), respectively. Data were analyzed using the Statistical Package for Social Sciences^®^ software (SPSS^®^ Inc., Chicago, IL, United States) version 22.0. A significance level of 0.05 was adopted.

## Results

### Sociodemographic characteristics of the participants

Of the 970 participants in the first phase, 333 completed the questionnaire again in the second phase, forming the sample for this study. Table 1 shows the characteristics of the participants. Most participants were women, had an undergraduate degree or higher, were divorced/widowed/single, lived with up to three people in a municipality with a population of up to 200,000, and had a *per capita* income ranging from $833.48 to $2,083.67. A significant change was observed only in household size between T1 and T2 (*p* < 0.05; Table 1).

**Table 1:** Characteristics of participants.

	n (%)
Gender	
Female	234 (70.3)
Male	99 (29.7)
Age	
18–27	83 (25.0)
28–38	104 (31.3)
39–49	63 (19.0)
50–60	59 (17.8)
61–71	23 (6.9)
City size	
<200,000	177 (53.3)
>200,000	155 (44.9)
Level of education	
Basic education	11 (3.3)
Undergraduate degree (incomplete or complete)	103 (31.0)
Graduate degree (incomplete or complete)	218 (65.7)
Marital status	
Divorced/widowed/single	178 (53.6)
Married/cohabiting/in a common-law union	154 (45.4)
Household size	
1	33 (9.9)
2	98 (29.5)
3 or more	201 (60.5)
*Per capita* income	
<$416.73	38 (11.4)
$416.74–$833.47	55 (16.6)
$833.48–$2083.67	135 (40.7)
>2083.68	104 (31.3)

*n = 333.*

### Consumption frequency of home-prepared meals and ready-to-eat food from food services

**Figure 1:**
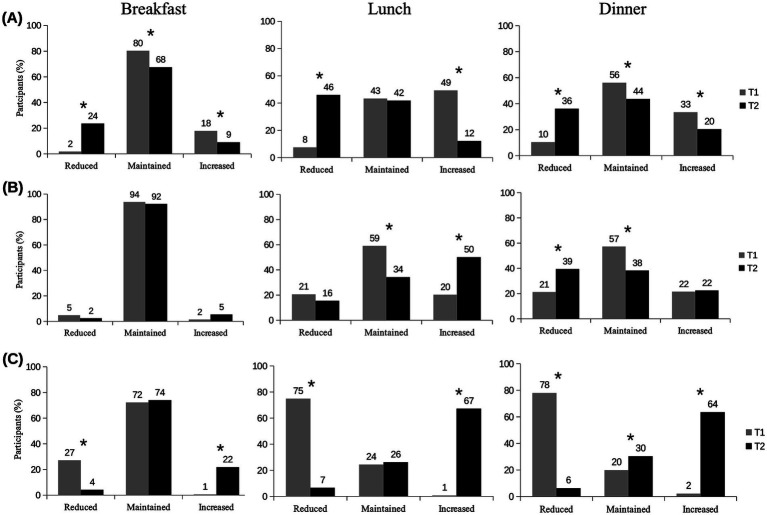
Change in the consumption frequency of home-prepared meals (A), ready-to-eat food purchased from off-site services (delivery, take-away, drive-thru) (B), and meals consumed at on-site services (C) in T1 (compared to T0) and in T2 (compared to T1). *n* = 335; *p < 0.01. McNemar Test; Off-site refers to delivery, take-away, drive-thru; on-site refers to consumption at food service establishments; T0 = pre-pandemic period; T1 = approximately 6 months after the start of the COVID-19 pandemic; T2 = approximately 18 months after T1.

Regarding the home-prepared meals, most participants maintained the same frequency of breakfast consumption in T1 and T2 (Figure 1A). However, some participants showed changes in the frequency of breakfast consumption, with an increase being more common in T1, while a reduction was more frequent in T2 (*p* < 0.01). For lunch and dinner, more participants reported changes, with increases also being more prevalent in T1 and reductions in T2 (p < 0.01).

The frequency of breakfast consumption from off-site services (delivery, take-away, drive-thru) was also maintained by most participants at both time points (Figure 1B). For lunch and dinner, more changes in consumption frequency were observed during the pandemic. In T2, 50% of participants increased their frequency of purchasing lunch off-site, and 39% reduced their off-site dinner purchases. These variations were only reported by around 20% of participants in T1 (*p* < 0.01; Figure 1B).

In terms of on-site meal consumption, the reduction in purchase frequency was more prevalent in T1 for breakfast, lunch, and dinner compared to T2 (Figure 1C). On the other hand, the frequency of on-site meal consumption increased mainly in T2 compared to T1 (*p* < 0.01).

### Food service safety and hygiene factors in consumer perception

At the beginning of the pandemic (T1), 45.9% of participants were afraid of contracting COVID-19 when ordering food for delivery, and 78.4% were afraid when dining on-site (Table 2). However, these fears decreased in T2, with only 21.0% of participants afraid of contracting COVID-19 from delivery and 52.0% from on-site dining (*p* < 0.001).

**Table 2:** Fear of contracting COVID-19 from food delivery (off-site consumption) and food service (on-site consumption) 6 months after the onset of the COVID-19 pandemic (T1), and 18 months after T1 (T2), in a Brazilian sample.

	n (%)		*p*-value^*^
	T1	T2	
Off-site consumption			
Yes	153 (45.9)	70 (21.0)	<0.001
Neutral	98 (29.4)	94 (28.2)	0.766
No	81 (24.3)	169 (50.8)	<0.01
On-site consumption			
Yes	261 (78.4)	173 (52.0)	<0.001
Neutral	50 (15.0)	80 (24.0)	0.001
No	21 (6.3)	80 (24.0)	<0.01

*n* = 333. *McNemar Test, *p* < 0.01.

Before the COVID-19 pandemic, most participants considered the price/sales/discounts, service time/quality, and menu/taste as the most important factors when selecting a food service for ready-to-eat meals (Table 3). During the pandemic, the menu/taste became the most important factor for participants in both T1 and T2. Hygiene and cleanliness of the establishment stopped being important to participants throughout the pandemic (*p* < 0.01). Regarding compliance with safety protocols, there was an initial increase in the percentage of participants who considered this important (from T0 to T1) for both delivery services and on-site dining, followed by a decrease in T2 (*p* < 0.01).

**Table 3:** Factors influencing food service selection, concerns and precautions taken, and payment methods used by a Brazilian sample when purchasing ready-to-eat food from food services before the COVID-19 pandemic and at two points during the pandemic (T1 and T2).

	n (%)			*p*-value^*^
	T0	T1	T2	
Food service selection factors for purchase of ready-to-eat food				
Affordable price, sales, and discounts/ Free delivery	168 (50.5)^a^	148 (44.4)^a^	234 (70.3)^b^	<0.001
Service quality and service/delivery time	199 (59.8)^a^	158 (47.4)^b^	0 (0.0)^c^	<0.001
Menu/taste	236 (70.9)^a^	181 (54.4)^b^	251 (75.4)^a^	<0.001
Location of the establishment	94 (28.2)^a^	75 (22.5)^a^	53 (15.9)^b^	<0.001
Hygiene and cleanliness of the establishment	142 (42.6)^a^	137 (41.1)^a^	0 (0.0)^b^	<0.001
Compliance with safety protocols during delivery (mask use, hand hygiene, safety distance between delivery workers and customers, among others)	96 (28.8)^a^	159 (47.7)^b^	0 (0.0)^c^	<0.001
Compliance with safety protocols in the restaurant (mask use, hand hygiene, environmental hygiene, safety distance between customers, among others)	30 (9.0)^a^	92 (27.6)^b^	55 (16.5)^c^	<0.001
Familiarity/popularity of the restaurant	76 (22.8)	71 (21.3)	69 (20.7)	0.717
Payment methods	52 (15.6)	53 (15.9)	54 (16.2)	0.971
Awareness of the financial difficulties of the establishment	22 (6.6)^a^	61 (18.3)^b^	22 (6.6)^a^	<0.001
No factor	5 (1.5)^a^	19 (5.7)^b^	3 (0.9)^a^	<0.001
Concerns regarding food delivery service purchases				
If the meal is prepared with hygiene practices	212 (63.7)^a^	147 (44.1)^b^	253 (76.0)^c^	<0.001
If the packaging material is cleanable	6 (1.8)^a^	102 (30.6)^b^	80 (24.0)^b^	<0.001
If the packaging is tamper-proof	25 (7.5)^a^	25 (7.5)^a^	106 (31.8)^b^	<0.001
How food delivery is conducted	10 (3.0)^a^	26 (7.8)^a^	108 (32.4)^b^	<0.001
No concerns	56 (16.8)^a^	19 (5.7)^b^	57 (17.1)^a^	<0.001
Precautions taken when purchasing meals via delivery service				
Verification of package integrity	185 (55.6)^a^	188 (56.5)^a^	229 (68.8)^b^	<0.001
Package cleanliness	73 (21.9)^a^	217 (65.2)^b^	143 (42.9)^c^	<0.001
Proper package disposal	138 (41.4)^a^	234 (70.3)^b^	150(45.0)^a^	<0.001
Heating the food prior to consumption	51 (15.3)^a^	83 (24.9)^b^	0 (0.0)^c^	<0.001
No precautions taken	43 (12.9)^a^	25 (7.5)^b^	33 (9.9)^ab^	0.016
Payment methods used in the purchase of ready-to-eat food				
Cash	141 (42.3)^a^	72 (21.6)^b^	60 (18.0)^b^	<0.001
Credit card	287 (86.2)^a^	258 (77.5)^b^	302 (90.7)^a^	<0.001
Mobile application	97 (29.1)^a^	135 (40.5)^b^	123 (36.9)^b^	<0.001
Payment bracelet	0 (0.0)	0 (0.0)	1 (0.3)	0.368
Bank transfer	10 (3.0)^a^	27 (8.1)^b^	29 (8.7)^b^	<0.001
Meal voucher^↓^	6 (1.8)	4 (1.2)	5 (1.5)	0.717
Food voucher^↑^	8 (2.4)^a^	9 (2.7)^a^	21 (6.3)^b^	<0.001

*n* = 333; T0 = pre-pandemic period; T1 = 6 months after the COVID-19 pandemic began; T2 = 18 months after T1. The sum of the percentages for each aspect evaluated is greater than 100 because participants could select more than one option. ↓Meal voucher refers to a predetermined amount of money provided in card form to employees for purchasing ready-to-eat meals. ↑Food voucher refers to a predetermined amount of money provided in card form to employees for purchasing foodstuffs. *Cochran’s Q test. ^a,b,c^Different lowercase letters in the same line indicate significant differences (*p* < 0.01).

Before the pandemic, the most commonly reported concern among participants when purchasing meals through delivery was whether the meal had been prepared using hygienic practices (Table 3). During the pandemic, this concern decreased in T1 but increased again to 76% in T2 (*p* < 0.01). Regarding packaging, concern about the material being washable increased similarly in both T1 and T2 compared to T0, while concern with tamper-proof packaging was more prevalent in T2 compared to T0 and T1. Similar results were observed regarding concerns about the food delivery method were more prevalent in T2 (p < 0.01).

After purchasing ready-to-eat meals through delivery, the most common care taken by participants in T0 was checking if the package had been tampered with and discarding it (Table 3). During the pandemic, there was an increase in the percentage of participants who took extra precautions with the packaging, such as checking for tampering (in T2 compared T0 and T1), cleaning the package (in T1 and T2 compared to T0), and disposing of it (in T1 compared to T0 and T2; *p* < 0.01). Similarly, care taken with food before consumption, such as heating, increased during the pandemic (more in T1 than T0), but this practice was no longer common in T2 (*p* < 0.01).

In terms of payment methods (Table 3), both pandemic moments saw a decrease in cash use compared to T0, while the use of bank transfers and mobile payment apps increased (*p* < 0.01). Credit cards were the most commonly used payment method both before and during the pandemic. Credit card use initially decreased in T1 compared to T0 but increased again in T2 to levels similar to T0 (*p* < 0.01), as shown in Table 3.

## Discussion

The prevalence of eating out has increased globally in recent decades (28), especially in Brazil (29). However, during the early months of the COVID-19 pandemic, restaurant operations were restricted for several months to minimize virus transmission (30), resulting in changes in food access and eating patterns (31). The first moment of the current study, T1, occurred around 6 months after the implementation of social distancing measures and restaurant closures in Brazil. As a result, the majority of participants reduced the frequency with which they consumed all on-site meals in T1.

Regarding ready-to-eat food purchased from off-site services, other studies have reported both a reduction and an increase in the use food delivery services during the early months of the pandemic (32–36). In the present study, nearly 60% of participants maintained their frequency of purchasing ready-to-eat meals, while only about 20% increased their frequency in T1 compared to T0. These results may be related to the fact that almost half of the participants were afraid of contracting COVID-19 when ordering food delivery, leading to an increase in the frequency of home cooking in T1. With social distancing measures, such as remote work and closure of non-essential establishments, a significant portion of the population spent more time at home (37), which may have contributed to the rise in home cooking observed in other studies (30, 38).

Shortly before T2, mask requirements for indoor settings were lifted in Brazil (39), and face-to-face dining was permitted, as long as hygiene and safety protocols were followed. These protocols included maintaining physical distance in lines, disinfecting tables and chairs after each customer, cleaning bathrooms more frequently, providing disposable gloves for self-service, offering individually packaged cutlery (40), requiring delivery workers and customers to wear face masks and gloves, using of contactless payment methods, and minimizing interactions between delivery workers and customers (23). By T2, approximately 83% of the Brazilian population had been vaccinated against COVID-19 (41).

In this context, there was an important decrease in the number of participants cooking lunch and dinner at home in T2. This finding can be attributed to the relaxation of restrictions and a reduction in the fear of contracting COVID-19 in food service settings by T2. Indeed, more than 60% of participants increased their on-site meal consumption in T2. The vaccine, the return to regular activities, and the removal of the requirement to wear masks during T2 may have created a false sense that the pandemic was over and there was no longer a risk of contracting COVID-19.

Regarding off-site services, 39% of participants reduced their dinner purchases. This may be partly due to the continued fear of contracting COVID (which persisted for 22% of participants), but was likely more related to the increased frequency of on-site dining in T2. In addition to convenience, dining at food establishments allows people to have a social experience (42).

On the other hand, half of the participants increased their lunch purchases from off-site services in T2. Off-site services offer consumers practicality and convenience (8), as well as access to a wide range of items and services. During the pandemic, delivery has become a critical lifeline for restaurants, and this rise in off-site meal consumption suggests that it will remain popular, or even grow, post-pandemic. From 2022 to 2029, the global food service industry is expected to grow at a compound annual growth rate (CAGR) of 10.76%, reaching a market value of 5,194.60 billion dollars (43).

When selecting a food service for ready-to-eat meals, the number of participants who prioritized compliance with safety protocols during delivery and on-site service increased in T1 but decreased in T2. Surprisingly, the selection of the establishment based on perceived hygiene and cleanliness did not increase among participants in T1, and this factor was not mentioned as a decisive criterion by any participant in T2. This may be because, during T1, on-site dining was less frequent compared to T0, and delivery customers were unable to assess the hygiene practices of an establishment. Once in-person dining resumed, participants may have felt more confident prioritizing other factors when choosing an establishment. It is important to highlight that participants were asked to select only the three most important factors when answering the questionnaire; therefore, the results reflect only their top priorities at the time.

Nevertheless, when they received food at home, participants remained concerned about hygiene practices (mainly in T2), packaging material (in both T1 and T2), safety (mainly in T2), and delivery methods (mainly in T2). Package-related precautions, such as cleaning and disposal, were more common during the pandemic than before it, although there were fluctuations between the two pandemic phases evaluated. Cleaning and disposing of packages became more prevalent in T1 but declined in T2. This may have been influenced by research indicating that the risk of COVID-19 transmission via surfaces is low. According to the US Centers for Disease Control and Prevention, the risk of contracting COVID-19 from a contaminated surface is less than 1% (44). Heating food before consumption also became more common as the virus spread. However, this precaution was abandoned by T2, likely due to widespread information disseminated by the press, clarifying that there is no evidence of COVID-19 transmission through food (45). On the other hand, checking for tampered packaging was a precaution adopted by most participants throughout the study and increased in T2. A survey performed in the United States found that 28% of 500 interviewed delivery drivers admitted to tasting the food of customers (46). Although there is no technical data on this topic in Brazil, experts speculate that the number may be higher (47). As food delivery has grown in demand since the pandemic began, cases of tampered food in deliveries may have increased, along with consumer concerns.

Regarding payment methods, the results of this study show a decline in cash usage and a surge in contactless methods during the pandemic, which was consistent with recommendations to reduce physical contact between customers and food service workers, thereby reducing the risk of COVID-19 transmission (23, 24).

This study has limitations, such as the need for internet access to complete the questionnaire. Additionally, the final sample is not representative of the entire Brazilian population, as it was primarily composed of younger individuals with higher education and income levels. Nonetheless, the study included participants from various regions of Brazil, with the majority residing in the Southeast. Another limitation was the fact that 34.2% of the participants dropped out between T1 and T2. Despite these limitations, this study has strengths. Longitudinal research on food service safety and hygiene from the perspective of consumers during the pandemic is limited, our findings contribute to a better understanding of changes in consumer behavior and perceptions during the COVID-19 pandemic. Future studies are needed to further investigate post-pandemic consumer behavior.

Our results show that the first wave of COVID-19 pandemic prompted changes in consumer behavior, including an increase in home-prepared meals, a rise in the number of participants prioritizing safety protocols during delivery and on-site service, greater concern about hygiene practices, packaging material, safety, delivery methods, and the use of contactless payment methods compared to the pre-pandemic period. These changes can be attributed to the fear of contracting COVID-19 when ordering food delivery, reported by nearly half of the participants, along with changes in daily life, such as remote work and closure of non-essential establishments. Conversely, with high vaccination rates and relaxation of social distancing measures, we observed that most participants increased their consumption of on-site meals and lunch deliveries, and concern for hygiene, safety, and delivery methods remained elevated. In contrast, concerns about compliance with safety protocols and fear of contracting COVID-19 decreased. Understanding consumer behavior and attitudes during health crises is essential to delivering effective and straightforward public health messages. Consumers play an important role in food safety since they can take precautions when eating out and apply social pressure to ensure safe practices in food services.

## Data Availability

The datasets presented in this article are not readily available because for ethical reasons, the data is protected with the researchers for 5 years. Requests to access the datasets should be directed to julianaliboredo@gmail.com.

## References

[1] Zanetta LD Dardaque Mucinhato RM Hakim MP Stedefeldt E Da Cunha DT. What motivates consumer food safety perceptions and beliefs? A scoping review in BRICS countries. Food Secur. (2022) 11:1–16. doi: 10.3390/foods11030432, PMID: 35159583 PMC8833883

[2] Zanetta LDA Hakim MP Stedefeldt E de Rosso VV Cunha LM Redmond EC. Consumer risk perceptions concerning different consequences of foodborne disease acquired from food consumed away from home: a case study in Brazil. Food Control. (2022) 133:108602. doi: 10.1016/j.foodcont.2021.108602

[3] de Andrade ML Rodrigues RR Antongiovanni N da Cunha DT. Knowledge and risk perceptions of foodborne disease by consumers and food handlers at restaurants with different food safety profiles. Food Res Int. (2019) 121:845–53. doi: 10.1016/j.foodres.2019.01.006, PMID: 31108817

[4] Machado Nardi VA Teixeira R Ladeira WJ de Oliveira SF. A meta-analytic review of food safety risk perception. Food Control. (2020) 112:107089. doi: 10.1016/j.foodcont.2020.107089

[5] Zanin LM Capriles DT Stedefeldt E. Knowledge, attitudes and practices of food handlers in food safety: an integrative review. Food Res Int. (2017) 100:53–62. doi: 10.1016/j.foodres.2017.07.042 28873718

[6] Hakim MP Zanetta LDA da Cunha DT. Should I stay, or should I go? Consumers’ perceived risk and intention to visit restaurants during the COVID-19 pandemic in Brazil. Food Res Int. (2021) 141:110152. doi: 10.1016/j.foodres.2021.110152, PMID: 33642018 PMC7834331

[7] Al Amin M Arefin MS Alam MR Ahammad T Hoque MR. Using Mobile food delivery applications during COVID-19 pandemic: an extended model of planned behavior. J Food Prod Mark. (2021) 27:105–26. doi: 10.1080/10454446.2021.1906817

[8] Gavilan D Balderas-Cejudo A Fernández-Lores S Martinez-Navarro G. Innovation in online food delivery: learnings from COVID-19. Int J Gastron Food Sci. (2021) 24:100330. doi: 10.1016/j.ijgfs.2021.100330, PMID: 34745390 PMC8562059

[9] Mehrolia S Alagarsamy S Solaikutty VM. Customers response to online food delivery services during COVID-19 outbreak using binary logistic regression. Int J Consum Stud. (2021) 45:396–408. doi: 10.1111/ijcs.12630, PMID: 33362434 PMC7753470

[10] Liboredo JC Amaral CAA Carvalho NC. Food delivery before and during the COVID-19 pandemic in Brazil. Nutr Food Sci. (2023) 53:301–18. doi: 10.1108/NFS-12-2021-0368

[11] Thomas MS Feng Y. Food handling practices in the era of COVID-19: a mixed-method longitudinal needs assessment of consumers in the United States. J Food Prot. (2021) 84:1176–87. doi: 10.4315/JFP-21-006, PMID: 33666666 PMC9906159

[12] Ferreira Rodrigues J dos Santos C Filho MT Aparecida de Oliveira LE Brandemburg Siman I De BA. Effect of the COVID-19 pandemic on food habits and perceptions: a study with Brazilians. Trends Food Sci Technol. (2021) 116:992–1001. doi: 10.1016/j.tifs.2021.09.005, PMID: 34539079 PMC8434886

[13] Abranches MV Oliveira TC José JFBS. Food service as public health space: health risks and challenges brought by the covid-19 pandemic. Interface: Commun, Health Educ. (2021) 25:1–16. doi: 10.1590/Interface.200654

[14] da Cunha DT. Improving food safety practices in the food service industry. Curr Opin Food Sci. (2021) 42:127–33. doi: 10.1016/J.COFS.2021.05.010

[15] Miyahira RF Matheus JRV. Food services in times of uncertainty: remodeling operations, changing trends, and looking into perspectives after the COVID-19 pandemic. Trends Food Sci Technol. (2022) 120:301–7. doi: 10.1016/J.TIFS.2022.01.005, PMID: 35035090 PMC8746399

[16] Vandenhaute H Gellynck X De Steur H. COVID-19 safety measures in the food service sector: consumers’ attitudes and transparency perceptions at three different stages of the pandemic. Food Secur. (2022) 11:1–20. doi: 10.3390/foods11060810, PMID: 35327233 PMC8947567

[17] Liboredo JC Amaral CAA Caldeira de Carvalho NC. Using of food service: changes in a Brazilian sample during the COVID-19 pandemic. Nutr Food Sci. (2024) 54:579–96. doi: 10.1108/NFS-06-2023-0129

[18] Liboredo JC Anastácio LR Ferreira LG Oliveira LA Della Lucia CM. Quarantine during COVID-19 outbreak: eating behavior, perceived stress, and their independently associated factors in a Brazilian sample. Front Nutr. (2021) 8:704619. doi: 10.3389/fnut.2021.704619, PMID: 34381806 PMC8349978

[19] Lee S Ham S. Food service industry in the era of COVID-19: trends and research implications. Nutr Res Pract. (2021) 15:S22–31. doi: 10.4162/NRP.2021.15.S1.S22, PMID: 34909130 PMC8636391

[20] Gaulion Consultoria para food service e Qualibest. (2020) Alimentação na pandemia: como a COVID-19 impacta os consumidores e os negócios em alimentação. Onda 2. Available at: http://dfreire.tempsite.ws/download/20200513_Alimentacao_na_Pandemia_Onda-2.pdf [Accessed August 19, 2021]

[21] Chua BL Karim S Lee S Han H. Customer restaurant choice: an empirical analysis of restaurant types and eating-out occasions. Int J Environ Res Public Health. (2020) 17. doi: 10.3390/ijerph17176276, PMID: 32872267 PMC7503372

[22] Medeiros CO Salay E. A review of food service selection factors important to the consumer. Food Public Health. (2013) 3, 76–190. doi: 10.5923/j.fph.20130304.02

[23] Nguyen THD Vu DC. Food delivery service during social distancing: proactively preventing or potentially spreading COVID-19? Disaster Med Public Health Prep. (2020) 14:e9–e10. doi: 10.1017/dmp.2020.135, PMID: 32367789 PMC7235311

[24] Rizou M Galanakis IM Aldawoud TMS Galanakis CM. Safety of foods, food supply chain and environment within the COVID-19 pandemic. Trends Food Sci Technol. (2020) 102:293–9. doi: 10.1016/j.tifs.2020.06.008, PMID: 32834502 PMC7295520

[25] Saad AT. Factors affecting online food delivery service in Bangladesh: an empirical study. Br Food J. (2021) 123:535–50. doi: 10.1108/BFJ-05-2020-0449

[26] Shahbaz M Bilal M Moiz A Zubair S Iqbal HMN. Food safety and COVID-19: precautionary measures to limit the spread of coronavirus at food service and retail sector. J Pure Appl Microbiol. (2020) 14:749–56. doi: 10.22207/JPAM.14.SPL1.12

[27] Zhao A Li Z Ke Y Huo S Ma Y Zhang Y. Dietary diversity among chinese residents during the COVID-19 outbreak and its associated factors. Nutrients. (2020) 12:1–13. doi: 10.3390/nu12061699, PMID: 32517210 PMC7352896

[28] Edwards JSA. The food service industry: eating out is more than just a meal. Food Qual Prefer. (2013) 27:223–9. doi: 10.1016/j.foodqual.2012.02.003

[29] Instituto Brasileiro de Geografia e Estatística Coordenação de Trabalho e Rendimento. Pesquisa de orçamentos familiares Pesquisa de orçamentos familiares, 2017-2018: primeiros resultados / Ministério da Economia, Instituto Brasileiro de Geografia e Estatística-IBGE, Diretoria de Pesquisas, Coordenação de Trabalho e Rendimento. análise do consumo alimentar pessoal no Brasil. 64 p. Rio de Janeiro: IBGE. Available at: https://biblioteca.ibge.gov.br/index.php/biblioteca-catalogo?view=detalhes&id=2101742 (Accessed April 4, 2024).

[30] Eftimov T Popovski G Petković M Seljak BK Kocev D. COVID-19 pandemic changes the food consumption patterns. Trends Food Sci Technol. (2020) 104:268–72. doi: 10.1016/j.tifs.2020.08.017, PMID: 32905099 PMC7462788

[31] Scarmozzino F Visioli F. Covid-19 and the subsequent lockdown modified dietary habits of almost half the population in an Italian sample. Food Secur. (2020) 9:1–8. doi: 10.3390/foods9050675, PMID: 32466106 PMC7278864

[32] Belarmino A Raab C Tang J Han W. Exploring the motivations to use online meal delivery platforms: before and during quarantine. Int J Hosp Manag. (2021) 96:102983. doi: 10.1016/j.ijhm.2021.102983

[33] Chevalier S. Brazil: Number of online food delivery users 2017–2024. (2020). Available at: https://www.statista.com/forecasts/1135443/brazil-online-food-delivery-users-by-segment [Accessed August 31, 2022]

[34] Cohen JFW Posluszny H Falbe J Mueller MP Gearhardt AN Leung CW. Restaurant dining during the COVID-19 pandemic among adults with low-income in the United States. Appetite. (2022) 173:105976. doi: 10.1016/j.appet.2022.105976, PMID: 35245643 PMC8885442

[35] Dezanetti T Quinaud RT Caraher M Jomori MM. Meal preparation and consumption before and during the COVID-19 pandemic: the relationship with cooking skills of Brazilian university students. Appetite. (2022) 175:106036. doi: 10.1016/j.appet.2022.106036, PMID: 35429579 PMC9007752

[36] O’Meara L Turner C Coitinho DC Oenema S. Consumer experiences of food environments during the Covid-19 pandemic: global insights from a rapid online survey of individuals from 119 countries. Glob Food Sec. (2022) 32:100594. doi: 10.1016/j.gfs.2021.100594, PMID: 34812406 PMC8598973

[37] De ATM Lua I. O trabalho mudou-se para casa: trabalho remoto no contexto da pandemia de COVID-19. Revista Brasileira de Saúde Ocupacional. (2021) 46, 1–11. doi: 10.1590/2317-6369000030720

[38] Steele EM Rauber F dos Santos CC Leite MA Gabe KT da Costa Louzada ML. Dietary changes in the NutriNet Brasil cohort during the covid-19 pandemic. Rev Saude Publica. (2020) 54:91. doi: 10.11606/S1518-8787.2020054002950, PMID: 32901755 PMC7454165

[39] BRASIL. Portaria Interministerial MTP/MS. Brazil: Diário Oficial da União (2022).

[40] ABRASEL. Associação Brasileira de Bares e Restaurantes. Guia para uma entrega segura em casa para todos. (2020). Available at: https://abrasel.com.br/noticias/noticias/empresas-de-tecnologia-se-unem-e-lancam-guia-de-prevencao-ao-covid-19/ (Accessed August 19, 2021).

[41] G1.Mapa da vacinação contra Covid-19 no Brasil. (2023). Available at: https://especiais.g1.globo.com/bemestar/vacina/2021/mapa-brasil-vacina-covid/ [Accessed April 10, 2024]

[42] Gustafsson I-B Öström Å Johansson J Mossberg L. The five aspects meal modelI. J. Foodserv. (2006) 17:84–93. doi: 10.1111/j.1745-4506.2006.00023.x

[43] Gitnux. Food service industry statistics. (2023). Available at: https://gitnux.org/food-service-industry-statistics/ [Accessed April 10, 2024]

[44] CDC. Centers for Disease Control and Prevention. Science brief: SARS-CoV-2 and surface (fomite) transmission for indoor community environments. (2021). Available at: https://archive.cdc.gov/www_cdc_gov/coronavirus/2019-ncov/more/science-and-research/surface-transmission.html [Accessed April 10, 2024] 34009771

[45] Anelich LECM Lues R Farber JM Parreira VR. SARS-CoV-2 and risk to food safety. Front Nutr. (2020) 7:580551. doi: 10.3389/fnut.2020.580551, PMID: 33224968 PMC7667249

[46] Fox5 New York. Survey finds 28 percent of delivery drivers have tasted food they’re delivering. (2019). Available at: https://www.fox5ny.com/news/survey-finds-28-percent-of-delivery-drivers-have-tasted-food-theyre-delivering [Accessed April 4, 2024]

[47] Food Safety Brazil. Violação de alimentos delivery – como assim? (2024). Available at: https://foodsafetybrazil.org/violacao-de-alimentos-delivery-como-assim/ [Accessed April 9, 2024]

