# Supplementary material for: Food service safety and hygiene factors: a longitudinal study on the Brazilian consumer perception
Source: Front Nutr. 2024 Oct 31;11:1416554. doi: 10.3389/fnut.2024.1416554 (PMC11560780; doi:10.3389/fnut.2024.1416554)
Supplement: Supplementary file 1 [file Data_Sheet_1.DOCX]

**Questionnaire**

**SECTION 1: Sociodemographic characteristics**

**Gender**

( ) Female

( ) Male

**Age ___________________________**

**City of residence: ____________________________**

**Level of education**

( ) Basic education

( ) Undergraduate degree (incomplete or complete)

( ) Graduate degree (incomplete or complete)

**Marital status**

( ) Divorced/widowed/single

( ) Married/cohabiting/in a common-law union

**Household size**

( ) 1

( ) 2

( ) 3 or more

**Per capita income**

( ) <$416.73

( ) $416.74-$833.47

( ) $833.48-$2083.67

( ) >2083.68

**SECTION 2: Frequency of home-prepared meals and on-site and off-site services (delivery, take-away, drive-thru)**

**How often did you eat home-cooked meals BEFORE the pandemic?**

|  | Never | Rarely | Once a week | 2–4 times/week | 5–6 times/week | Once a day |
| --- | --- | --- | --- | --- | --- | --- |
| Breakfast | ( ) | ( ) | ( ) | ( ) | ( ) | ( ) |
|  |  |  |  |  |  |  |
| Lunch | ( ) | ( ) | ( ) | ( ) | ( ) | ( ) |
|  |  |  |  |  |  |  |
| Dinner | ( ) | ( ) | ( ) | ( ) | ( ) | ( ) |
|  |  |  |  |  |  |  |

**How often did you eat home-cooked meals DURING the pandemic?**

|  | Never | Rarely | Once a week | 2–4 times/week | 5–6 times/week | Once a day |
| --- | --- | --- | --- | --- | --- | --- |
| Breakfast | ( ) | ( ) | ( ) | ( ) | ( ) | ( ) |
|  |  |  |  |  |  |  |
| Lunch | ( ) | ( ) | ( ) | ( ) | ( ) | ( ) |
|  |  |  |  |  |  |  |
| Dinner | ( ) | ( ) | ( ) | ( ) | ( ) | ( ) |
|  |  |  |  |  |  |  |

**How often did you order the meals below for delivery, take-out or drive-thru BEFORE the pandemic?**

|  | Never | Rarely | Once a week | 2–4 times/week | 5–6 times/week | Once a day |
| --- | --- | --- | --- | --- | --- | --- |
| Breakfast | ( ) | ( ) | ( ) | ( ) | ( ) | ( ) |
|  |  |  |  |  |  |  |
| Lunch | ( ) | ( ) | ( ) | ( ) | ( ) | ( ) |
|  |  |  |  |  |  |  |
| Dinner | ( ) | ( ) | ( ) | ( ) | ( ) | ( ) |
|  |  |  |  |  |  |  |

**How often did you order the meals below for delivery, take-out or drive-thru DURING the pandemic?**

|  | Never | Rarely | Once a week | 2–4 times/week | 5–6 times/week | Once a day |
| --- | --- | --- | --- | --- | --- | --- |
| Breakfast | ( ) | ( ) | ( ) | ( ) | ( ) | ( ) |
|  |  |  |  |  |  |  |
| Lunch | ( ) | ( ) | ( ) | ( ) | ( ) | ( ) |
|  |  |  |  |  |  |  |
| Dinner | ( ) | ( ) | ( ) | ( ) | ( ) | ( ) |
|  |  |  |  |  |  |  |

**How often did you go to restaurants, cafes, bars and others and eat on-site BEFORE the pandemic?**

|  | Never | Rarely | Once a week | 2–4 times/week | 5–6 times/week | Once a day |
| --- | --- | --- | --- | --- | --- | --- |
| Breakfast | ( ) | ( ) | ( ) | ( ) | ( ) | ( ) |
|  |  |  |  |  |  |  |
| Lunch | ( ) | ( ) | ( ) | ( ) | ( ) | ( ) |
|  |  |  |  |  |  |  |
| Dinner | ( ) | ( ) | ( ) | ( ) | ( ) | ( ) |
|  |  |  |  |  |  |  |

**How often did you go to restaurants, cafes, bars and others and eat on-site DURING the pandemic?**

|  | Never | Rarely | Once a week | 2–4 times/week | 5–6 times/week | Once a day |
| --- | --- | --- | --- | --- | --- | --- |
| Breakfast | ( ) | ( ) | ( ) | ( ) | ( ) | ( ) |
|  |  |  |  |  |  |  |
| Lunch | ( ) | ( ) | ( ) | ( ) | ( ) | ( ) |
|  |  |  |  |  |  |  |
| Dinner | ( ) | ( ) | ( ) | ( ) | ( ) | ( ) |
|  |  |  |  |  |  |  |

**SECTION 3: Food service safety and hygiene factors in consumer perception**

**What are your mean concern related to purchasing meals from food delivery service (from restaurants, cafeterias, bars, and others)?**

|  | Before the pandemic | During the pandemic |
| --- | --- | --- |
| If the meal is prepared with hygiene practices | ( ) | ( ) |
| If the packaging material is cleanable | ( ) | ( ) |
| If the packaging is tamper-proof | ( ) | ( ) |
| How food delivery is conducted | ( ) | ( ) |
| No concerns | ( ) | ( ) |

**Select the payment methods used in the purchase of ready-to-eat food**

*(Check as many options as needed)*

|  | Before the pandemic | During the pandemic |
| --- | --- | --- |
| Cash | ( ) | ( ) |
| Credit card | ( ) | ( ) |
| Mobile application | ( ) | ( ) |
| Payment bracelet | ( ) | ( ) |
| Bank transfer | ( ) | ( ) |
| Meal voucher | ( ) | ( ) |
| Food voucher | ( ) | ( ) |

**What food service selection factors do you consider when purchasing ready-to-eat food?**

*(Check the 3 most important factors)*

|  | Before the pandemic | During the pandemic |
| --- | --- | --- |
| Affordable price, sales, and discounts/ Free delivery | ( ) | ( ) |
| Service quality and service/delivery time | ( ) | ( ) |
| Menu/taste | ( ) | ( ) |
| Location of the establishment | ( ) | ( ) |
| Hygiene and cleanliness of the  establishment | ( ) | ( ) |
| Compliance with safety protocols  during delivery (mask use, hand hygiene, safety distance between delivery workers and customers, among others) | ( ) | ( ) |
| Compliance with safety protocols in the restaurant (mask use, hand hygiene, environmental hygiene, safety distance between customers, among others) | ( ) | ( ) |
| Familiarity/popularity of the restaurant | ( ) | ( ) |
| Payment methods | ( ) | ( ) |
| Awareness of the financial  difficulties of the establishment | ( ) | ( ) |
| No factor | ( ) | ( ) |

**What precautions do you take when purchasing meals via food delivery service?**

*(Check as many options as needed)*

|  | Before the pandemic | During the pandemic |
| --- | --- | --- |
| Verification of package integrity | ( ) | ( ) |
| Packaging cleanliness | ( ) | ( ) |
| Proper package disposal | ( ) | ( ) |
| Heating the food prior to consumption | ( ) | ( ) |
| No precautions taken | ( ) | ( ) |

**I am afraid of contracting COVID-19 from food delivery services.**

1- strongly disagree; 2- disagree; 3- Neither agree nor disagree; 4- agree; 5- strongly agree

**I am afraid of contracting COVID-19 by going to restaurants, cafes, bars and others.**

1. strongly disagree; 2- disagree; 3- Neither agree nor disagree; 4- agree; 5- strongly agree

***Note:*** The same questionnaire was used in the second phase (T2) of the study. The wording of the questions was changed to ask about the present time of data collection, but all the answer options remained the same.
